# Predicting potentially pathogenic effects of *h*RPE65 missense mutations: a computational strategy based on molecular dynamics simulations

**DOI:** 10.1080/14756366.2022.2090547

**Published:** 2022-06-21

**Authors:** Giulio Poli, Ivana Barravecchia, Gian Carlo Demontis, Andrea Sodi, Alessandro Saba, Stanislao Rizzo, Marco Macchia, Tiziano Tuccinardi

**Affiliations:** a Department of Pharmacy, University of Pisa, Pisa, Italy; b Institute of Life Sciences, Scuola Superiore Sant’Anna, Pisa, Italy; c Department of Neurosciences, Psychology, Drug Research and Child Health Eye Clinic, University of Florence, AOU Careggi, Florence, Italy; d Department of Surgical Pathology, Molecular Medicine and of the Critical Area, University of Pisa, Pisa, Italy; e Ophthalmology Unit, Fondazione Policlinico Universitario A. Gemelli IRCCS, Rome, Italy; f Catholic University Sacro Cuore, Rome, Italy; g Consiglio Nazionale delle Ricerche, Istituto di Neuroscienze, Pisa, Italy

**Keywords:** RPE65, variant of uncertain significance, molecular dynamics, missense mutations

## Abstract

The human retinal pigment epithelium-specific 65-kDa protein (*h*RPE65) plays a crucial role within the retinoid visual cycle and several mutations affecting either its expression level or its enzymatic function are associated with inherited retinal diseases such as Retinitis Pigmentosa. The gene therapy product voretigene neparvovec (Luxturna) has been recently approved for treating hereditary retinal dystrophies; however, the treatment is currently accessible only to patients presenting confirmed biallelic mutations that severely impair *h*RPE65 function, and many reported *h*RPE65 missense mutations lack sufficient evidences for proving their pathogenicity. In this context, we developed a computational approach aimed at evaluating the potential pathogenic effect of *h*RPE65 missense variants located on the dimerisation domain of the protein. The protocol evaluates how mutations may affect folding and conformation stability of this protein region, potentially helping clinicians to evaluate the eligibility for gene therapy of patients diagnosed with this type of *h*RPE65 variant of uncertain significance.

## Introduction

Photon capture by opsins, a group of proteins belonging to the G protein-coupled receptor superfamily, starts visual perception in rod and cone photoreceptors of the vertebrate retina. Rod and cone opsins require 11-cis-retinaldehyde (11-cis-RAL) as a chromophore to operate as light sensors^1^. Photon absorption triggers 11-cis-RAL rapid isomerisation to all-trans retinaldehyde (all-trans-RAL), eventually dissociating into opsin and free all-trans-RAL^2^. Visual pigment regeneration represents a critical step in keeping photoreceptors responsive to light. For this purpose, the all-trans-RAL must be efficiently recycled via isomerisation in all-cis-RAL by a multistep process termed retinoid visual cycle, which involves several enzymatic steps^3^. The all-trans-RAL is initially reduced to all-trans-retinol (all-trans-ROL), the substrate for lecithin:retinol acyltransferase (LRAT), which catalyses the esterification of all-trans-ROL to all-trans-retinyl esters (all-trans-RE) in the retinal pigment epithelium (RPE). The all-trans-RE are then isomerised and hydrolysed to 11-cis-retinol (11-cis-ROL) by the retinal pigment epithelium-specific 65-kDa protein (RPE65), an isomerohydrolase enzyme^4^. Finally, 11-cis-ROL oxidation to 11-cis-retinal and its export from RPE cells to photoreceptors regenerates the visual pigment.

RPE65, identified as the retinoid isomerase in the visual cycle, is abundantly expressed in RPE cells and shuttles between the cytoplasm (low enzymatic activity) and the endoplasmic reticulum (high enzymatic activity)^5^. Previous studies have indicated membrane association of RPE65 as essential for its enzymatic activity^6^. RPE65 is highly conserved across all vertebrates, presenting high homology to mammalian Beta-carotene 15,15′ and cyanobacteria apo-carotene oxygenase^7^. The fully-active RPE65 protein is a dimer of two symmetrical, enzymatically independent subunits and is an iron-dependent enzyme in which four histidine residues coordinate a Fe^2+^ cation^8^. RPE65 has been demonstrated to bind stereospecifically all-trans-RE with high affinity to catalyse its isomerisation and hydrolysis to 11-cis-ROL. The RPE65 reaction is the rate-limiting step in the visual cycle, because it catalyses the regeneration of visual pigment that initiates the vision process. The crucial role of RPE65 in the visual cycle has been proved in a model of genetically engineered RPE65 knock-out mice model. RPE65^-/-^ animals did not convert all-trans-RE to generate 11-cis-ROL resulting in overaccumulation of all-trans-RE in the RPE; consequently, the photoreceptors in these mice became severely insensitive to light, and Rpe65^-/-^ mice underwent a slow and progressive loss of photoreceptors^9^.

A high number of pathogenic variants have been identified in humans since the RPE65 gene discovery. Many of these variants introduce a missense or nonsense mutation that affects either protein expression level or its enzymatic function and are associated with a spectrum of inherited retinal diseases ranging from Leber Congenital Amaurosis (LCA), Severe Early Childhood Onset Retinal Dystrophy (SECORD) and Retinitis Pigmentosa (RP)^10^. These diseases are associated with a progressive degeneration of retinal photoreceptors and a very severe visual loss. They usually affect young patients, in the midst of their family and work life, thus representing a severe human, social and economic burden for society. In December 2017, the Food and Drug Administration approved voretigene neparvovec (Luxturna) as the first gene therapy product for hereditary retinal dystrophy^11–13^. This gene therapy is based on a recombinant adeno-associated virus 2 (AVV2) expressing human wild-type RPE65, rescuing enzyme activity in RPE to improve vision in patients^14^^,^^15^. However, as established by the European Medicines Agency (EMA) and by the Italian Drug Agency (Agenzia Italiana del Farmaco, AIFA), voretigene neparvovec is currently accessible only to patients presenting confirmed RPE65 biallelic mutations^16^. The limitations to the access to the treatment are justified by the high cost of the drug, the organisation burden and the risks associated with surgery. Only if the patient carries two pathogenic RPE65 mutations we can reasonably assume that they represent the main cause of the patient retinal degeneration and that all troubles and risks associated with voretigene neparvovec delivery are really justified. Due to the wide range of RPE65 variants, recognising disease-causing variants from those of uncertain significance (VUS) represents a critical issue. Although variants leading to truncated or non-encoded protein may be expected to be pathogenic, the pathogenicity of missense mutations can be more challenging to predict. At present, more than 100 missense mutations lacking a clear pathogenicity classification or classified as VUS have been reported for RPE65 in public databases^17–20^. Considering that patients with RPE65 variants identified as pathogenic may access gene therapy at an early stage of the disease before reaching an advanced photoreceptor degeneration stage, the development of new methodological approaches able to decipher the potential pathogenicity of specific RPE65 VUS presented by patients may prove useful for the evaluation of their eligibility for gene therapy^13^.

In this article, we present an innovative *in silico* protocol developed to address the issue of patients diagnosed with an RPE65 VUS. In particular, we focussed on the impact of missense mutations located within the dimerisation site of RPE65, examining how these mutations could affect the folding and conformation stability of this structural region of the protein, essential for subcellular localisation and therefore protein stability, expression and enzymatic activity.

## Materials and methods

### Protein structure modelling

The X-ray structure of bovine RPE65 (PDB code 3FSN)^21^, which shows 98.5% of sequence identity with human RPE65 (*h*RPE65), was used for this study. The structural portion of the protein corresponding to the dimer-mediating sequence (DMS) and its surrounding residues (included in a shell of about 20 Å from the DMS residues) were isolated from the rest of the structure, thus obtaining a protein system including residues 227–255, 285–337 and 360–418, for a total of 141 protein residues. The few non-conserved residues included in the structural portion of the protein used for this study were mutated and the orientation of their side chains was automatically optimised using Modeller software^22^, thus obtaining a monomeric wilt-type (WT) DMS-focussed *h*RPE65 model. The monomeric models of the two *h*RPE65 variants, A393E and N302I, were obtained by mutating the proper residues of the WT *h*RPE65 monomeric model and automatically optimising the side chains of the mutated residues using Modeller.

The three DMS-focussed dimeric models, corresponding to the WT-WT, A393E-WT and N302I-WT dimers, were obtained by combining the previously created monomeric systems based on the reference X-ray structure of bovine RPE65 (PDB code 3FSN), i.e. superimposing the proper couples of monomeric structures to the crystallographic RPE65 dimer.

### Molecular dynamics simulations

All simulations were performed using AMBER, version 20^23^, using the ff14SB force field. The solute was placed in a rectangular parallelepiped water box, by using TIP3P explicit solvent model and solvated with an 8.0 Å water cap. Sodium ions were added as counterions to neutralise the systems. Before molecular dynamics (MD) simulations, the whole systems were energy minimised using a two-stage protocol. In the first stage, 5000 steps of steepest descent (SD) followed by conjugate gradient (CG) algorithms were performed for the exclusive minimisation of the solvent, since a harmonic potential of 100 kcal/(mol·Å^2^) was applied to all solute atoms. In the second stage, 5000 additional steps of SD/CG were used to minimise the whole system, until a convergence of 0.05 kcal/(mol·Å^2^), imposing a harmonic constraint of 10 kcal/(mol·Å^2^) only on the protein α carbons. The minimised systems were used as starting conformations for the MD simulations, which were performed using Particle Mesh Ewald (PME) electrostatics, periodic boundary conditions and a cut-off of 10 Å for the non-bonded interactions. SHAKE algorithm was employed to keep all bonds involving hydrogen atoms rigid. A constant volume periodic boundary MD was carried out for 0.5 ns, during which the temperature of the systems was raised from 0 to 300 K. The systems were then pre-equilibrated through 3 ns of constant pressure simulation, using the Langevin thermostat, in order to maintain the temperature at the constant value of 300 K. During these first two MD stages, all the protein α carbons were restrained with a harmonic potential of 10 kcal/(mol·Å^2^) and the simulation was performed using a time step of 2.0 fs. An additional constant pressure MD stage of 10 ns was then performed for equilibrating the system using the hydrogen mass repartition (HMR) scheme^24^ and a time step of 4.0 fs. All the protein α carbons were restrained with the same harmonic potential of 10 kcal/(mol·Å^2^). Finally, a production stage corresponding to 5 µs of constant pressure MD simulation was performed using the HMR scheme and a time step of 4.0 fs. During the production stage, the α carbons of only 56 out of the 141 protein residues belonging to the structural portion of RPE65 simulated in the system were subjected to the harmonic restraint of 10 kcal/(mol·Å^2^); this way, the DMS domain and the surrounding protein residues of the adjacent β-sheets were kept totally free to move during the MD and only the external residues were restrained.

The MD replica of the wild-type system was performed starting from the pre-equilibrated wild-type system, which was then subjected to the 10 ns equilibration and the 5 µs production stages without applying the HMR scheme and using a time step of 2.0 fs, while maintaining all other simulation parameters.

The MD simulations of the dimeric models were carried out using the same system preparation, minimisation and simulation protocols, as well as the same parameters, employed for the monomeric models. The only exception concerned the MD production stage performed using the HMR scheme, which was limited to a simulation length of 3 µs, during which the harmonic restraint of 10 kcal/(mol·Å^2^) was applied symmetrically to the same 56 α carbons of both monomers, as performed for the monomeric systems. The MD trajectories corresponding to the production stage of all systems were analysed using the cpptraj program^25^ implemented in Amber 20.

## Results and discussion

**Figure 1.**
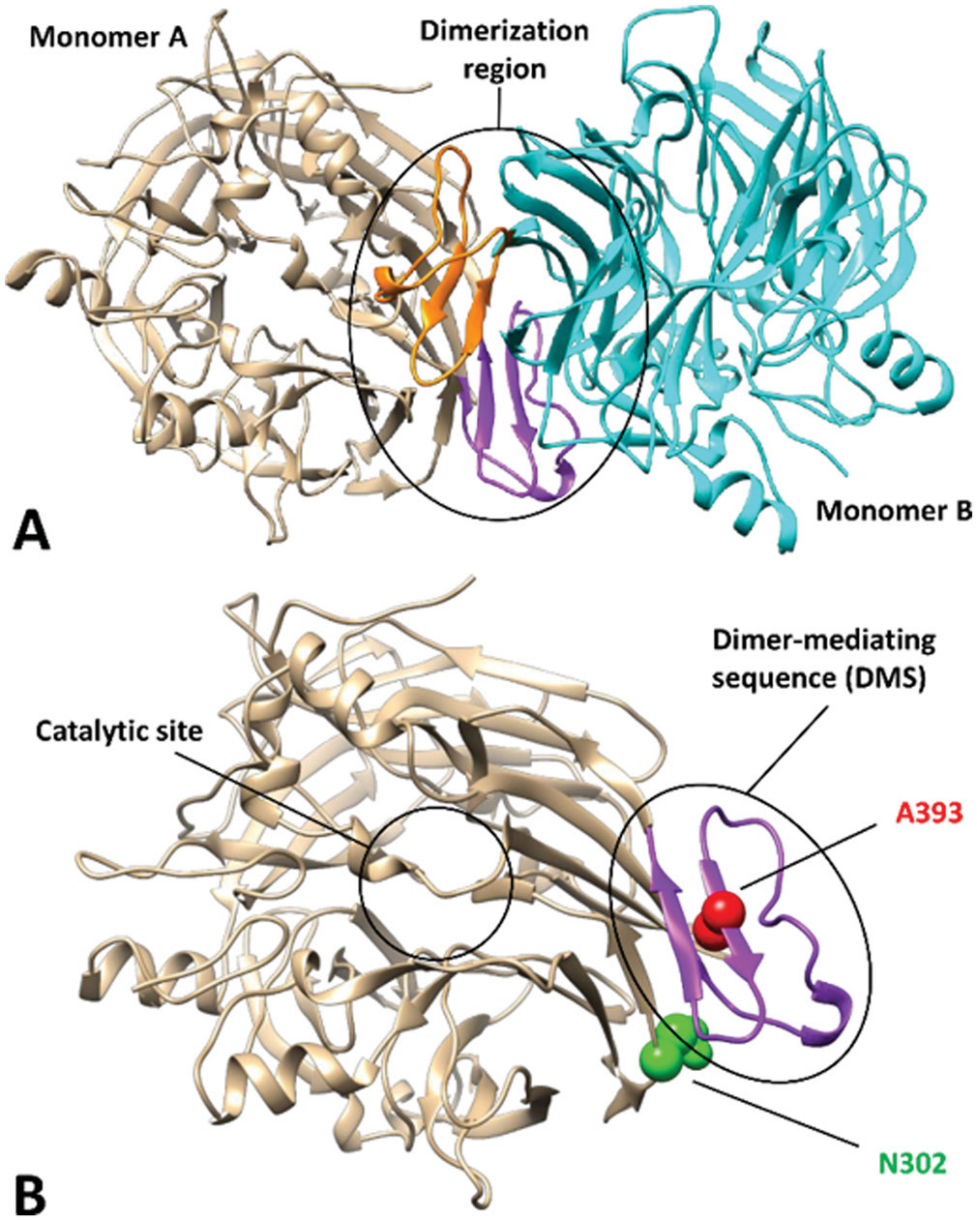
X-ray structure of bovine RPE65 (PDB code 3FSN). (A) The two monomers of the homodimer are shown in beige and cyan, with the corresponding dimer-mediating regions (DMS) coloured purple and orange, respectively. (B) A close up of a single monomer is shown, highlighting the distance of the catalytic site from the DMS, in which the two residues A393 and N302, associated with known missense mutations, are shown.

The final goal of our study was the development of an *in silico* strategy, based on MD simulations, allowing the evaluation of the potential pathogenic effect of specific missense mutations of *h*RPE65 associated with an insufficient amount of evidences for suggesting either their (likely) pathogenic or benign effect. For this purpose, we aimed at identifying a reliable MD protocol able to discriminate pathogenic or likely pathogenic mutations from benign or likely benign variants. We thus first analysed the literature in order to find information about known *h*RPE65 variants for identifying template mutations to be used as a reference for our protocol. We then focussed our attention on a particular domain of *h*RPE65 involved in its dimerisation. *h*RPE65 is regarded as a monotopic membrane protein existing as a homodimer *in vivo*^8^. X-ray studies involving bovine RPE65, which shares 98.5% identity with the human isoform, showed that the main portion of the protein involved in its dimerisation includes a sequence of about 40 residues mediating most of the interactions between the two monomers, thus referred to as dimer-mediating sequence (DMS). The sequence originates from an extension of the third blade of the β-propeller structure characterising the enzyme, primarily constituted by an antiparallel β-sheet and a loop connecting it to the core structure of the protein (Figure 1(A)). Interestingly, we found that a residue located within the DMS (A393) was associated to a missense mutation (A393E) found in patients affected by Leber congenital amaurosis (LCA) and reported to be likely connected to the pathogenic phenotypes^26–28^. Conversely, a residue not actually included in the DMS but adjacent to both the loop and the β-sheet of the DMS (N302) was found to be associated to a missense mutation (N302I) that can be considered as benign based on experimental evidences (Figure 1(B)). In fact, the effect of such mutation on *h*RPE65 hydrolase activity was experimentally evaluated and showed to be non-deleterious. In particular, the mutated residue I302 corresponds to the homolog residue of chicken RPE65, known to be endowed with a higher hydrolase activity with respect to the human isoform; consistently, N302I mutation demonstrated to slightly improve the catalytic activity of the human enzyme (about 125% of activity compared to wild type)^29^.

**Figure 2.**
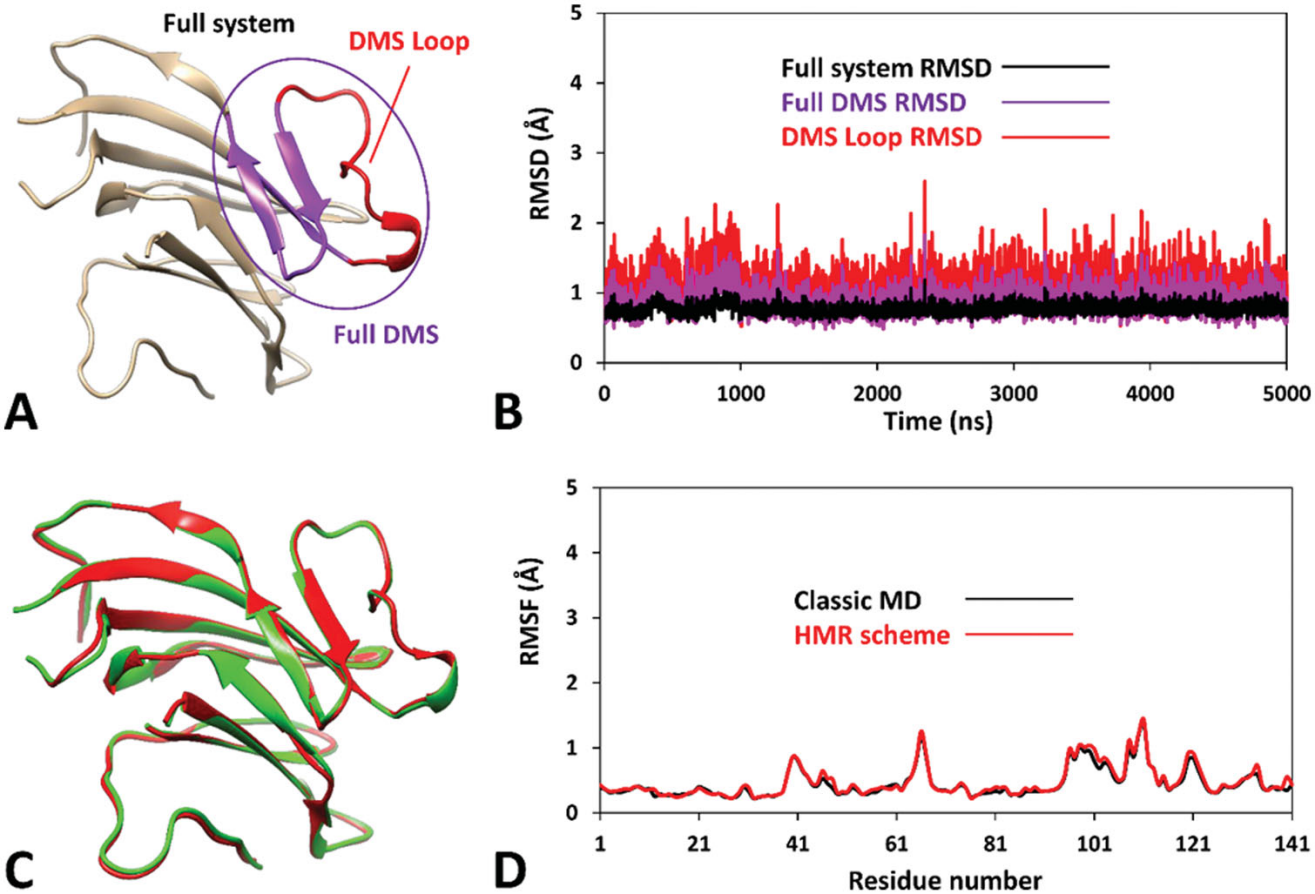
Results of the MD simulation studies performed on the wild-type *h*RPE65 system (A): (B) RMSD results obtained after 5 µs of MD simulation using the HMR scheme; (C) average structures of wild-type *h*RPE65 system obtained from the last 2 µs of MD simulation using classic (red) and HMR (green) approaches; (D) RMSF results obtained after 5 µs of MD using classic and HMR approaches.

Based on these considerations, we focussed on the development of an MD-based protocol for the prediction of the potential pathogenicity of *h*RPE65 missense mutations localised within the dimerisation domain, including the DMS and the surrounding regions. Precisely, we aimed at evaluating the potential deleterious effect on local folding and conformation stability of mutations located in such structural region of the protein, using the known variants A393E and N302I as a reference of likely harmful and benign mutations, respectively. As a first step, we assessed the reliability of a *h*RPE65 model, based on the X-ray structure of bovine RPE65 (PDB code 3FSN)^21^, including only the protein dimerisation domain (see Materials and Methods for details). Since this region constitutes a well-defined structural portion of the protein, located in a peripheral zone with respect to the catalytic site (Figure 1(B)), we envisioned that it could be analysed almost independently from the rest of the protein using a DMS-focussed system, thus obtaining a considerable increase in MD simulation speed. For this reason, our system included only the DMS and a small adjacent portion of the protein structure (Figure 2(A)), in which the outer and terminal residues were subjected to position restraints to serve as anchoring points of the system, while all other residues were left totally free to move during the MD, for allowing potential conformational changes. The generated system was then subjected to a 5 µs MD simulation protocol using the hydrogen mass repartitioning (HMR) scheme, which redistributes part of the mass of the heavy atoms connected to hydrogens into the bonded hydrogens, in order to further boost the speed of the MD by increasing the simulation time step (see Materials and Methods for details). Moreover, a replica of the simulation using the classic MD approach was also performed in order to confirm the reliability of the HMR approach for the *h*RPE65 DMS-focussed system. Figure 2(B), illustrates the results obtained for the HMR-based MD in terms of root-mean-square deviation (RMSD) of the protein α carbons, for both the full system and its key portions, which all presented remarkable stability, since even the most flexible sequence (the DMS loop) showed an average RMSD during the whole MD around 1.0 Å. Moreover, the results were fully comparable to those produced by the classic MD approach, as shown by the superposition of the minimised average structures obtained from the last 2 µs of both simulations (Figure 2(C)), which were found to be fully comparable both to each other and to the starting conformation of the system. Moreover, the analysis of the root-mean-square fluctuation (RMSF) of all protein α carbons during the MD further confirmed both the stability of the system, whose highest fluctuations were found to be lower than 1.5 Å, and the reliability of the HMR-scheme compared to the classic MD approach, since the deviation between the RMSF values obtained for the two simulations did not exceed 0.06 Å (Figure 2(D)). These results confirmed that the HMR scheme could be safely applied to boost the simulation speed without altering the results with respect to a classic MD approach.

**Figure 3.**
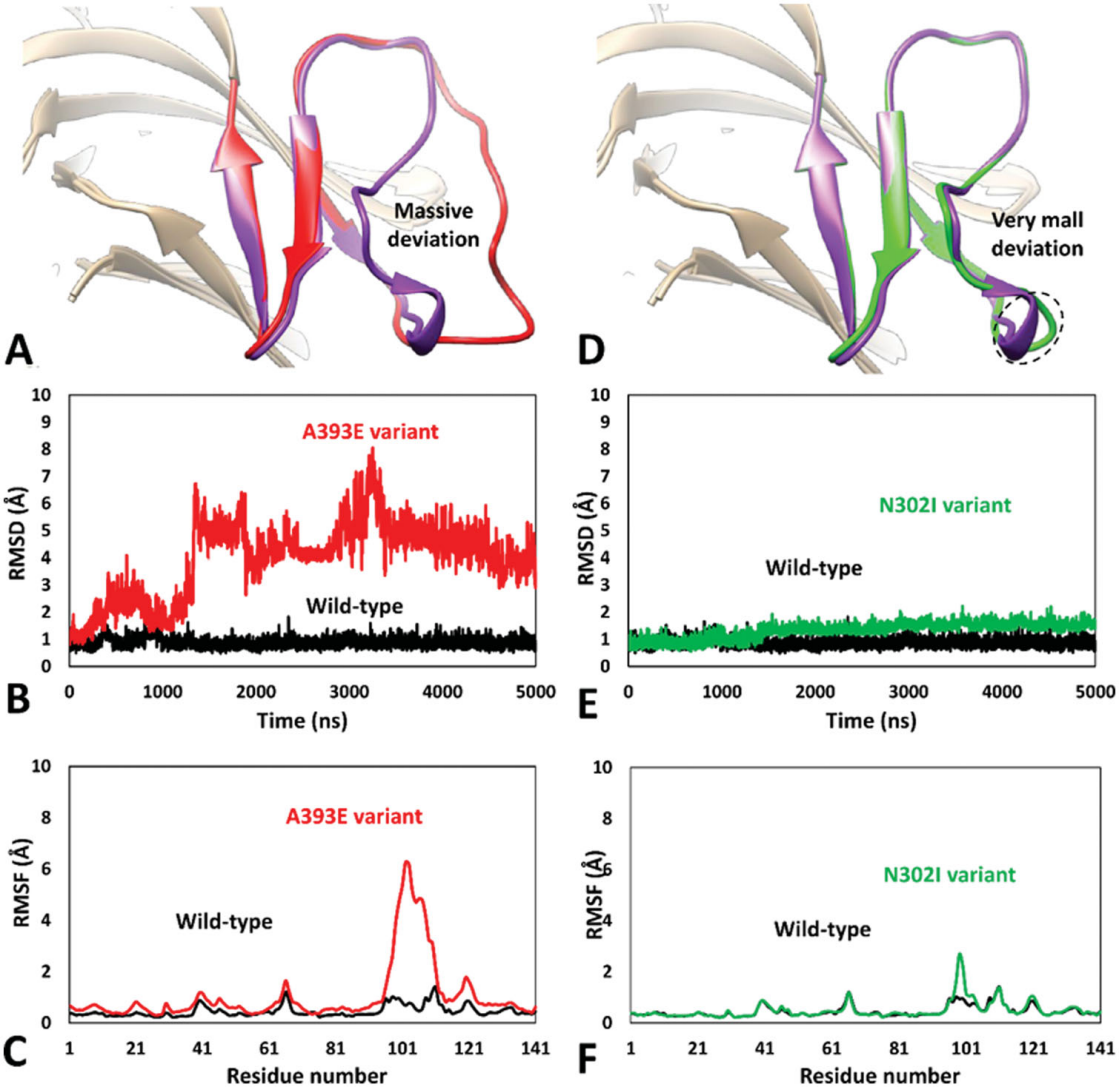
MD results obtained for A393E and N302I variants of *h*RPE65. (A) Average structures of A393E and WT systems obtained from the last 2 µs of MD simulation; the DMS of the two systems is respectively coloured red and purple. (B) RMSD of the DMS α carbons during 5 µs of MD simulation obtained for A393E system compared to WT. (C) RMSF of the whole A393E system α carbons compared to WT. (D) Average structures of N302I and WT systems obtained from the last 2 µs of MD simulation; the DMS of the two systems is respectively coloured green and purple. (E) RMSD of the DMS α carbons during 5 µs of MD simulation obtained for N302I system compared to WT. (F) RMSF of the whole N302I system α carbons compared to WT.

Encouraged by these results, we aimed at analysing the impact of A393E and N302I missense mutations on *h*RPE65 dimerisation region by using the validated MD protocol. For this reason, models of the two variants were generated and subjected to 5 µs of MD simulation using the HMR scheme. The results obtained for A393E variant highlighted a marked deleterious impact of the mutation on the DMS stability. In fact, the bigger side chain of E393 compared to that of the wild type (WT) residue, determined a destabilising effect on the DMS conformation, whose loop was rapidly pushed away from the β-sheet. In particular, after about 1.3 μs of simulation, the DMS loop underwent a considerable unfolding process, assuming multiple non-native conformations, during the rest of the MD, associated with a massive average deviation compared to the WT system (Figure 3(A)). This was also demonstrated by the analysis of the RMSD of the DMS α-carbons, which showed a rapid increase to values around 5 Å with multiple peaks, in contrast to the values obtained for the WT system that steadily remained around 1.0 Å (Figure 3(B)). Moreover, the analysis of the RSMF of the whole system α carbons showed fluctuations of up to 6.5 Å, indeed associated to the residues of the DMS loop (Figure 3(C)). These results clearly support the idea that the missense mutation A393E has a deleterious impact on *h*RPE65 folding and conformation stability, which may affect protein dimerisation and justify a pathogenic effect, in agreement with literature reports. These result, can thus be considered as a validation of the MD protocol in revealing potentially deleterious structure-based effects of *h*RPE65 missense mutations associated with potentially pathogenic phenotypes. On the contrary, the MD study performed on N302I variant of *h*RPE65 did not show a significant impact of the mutation on the conformation stability and folding of the analysed system (Figure 3(D)). In fact, the RMSD analysis revealed that the DMS conformation was perfectly maintained for the first 1.5 μs of simulation and only a very small increase in the average RMSD (to a value of about 1.6 Å), compared with the WT system, was observed for the remaining 3.5 μs of MD (Figure 3(E)). The RMSF analysis showed a slightly increased fluctuation of only few residues located within the DMS loop, corresponding to D375, K376 and A377, while the conformation of the rest of the DMS sequence was essentially unchanged with respect to the WT system (Figure 3(F)). Overall, these results suggest that the missense mutation N302I does not determine a deleterious effect on protein folding and stability, especially if the negligible conformational change observed for N302I variant (Figure 3(D)) is compared with the unfolding of the DMS, characterised by the loss of the native conformation of the DMS loop, shown by A393E variant (Figure 3(A)). This is consistent with the experimental data demonstrating a non-detrimental effect of N302I mutation on RPE65 activity and thus represents a validation of the MD protocol in confirming the harmless structural impact of non-pathogenic RPE65 missense mutations experimentally determined as neutral and/or benign.

**Figure 4.**
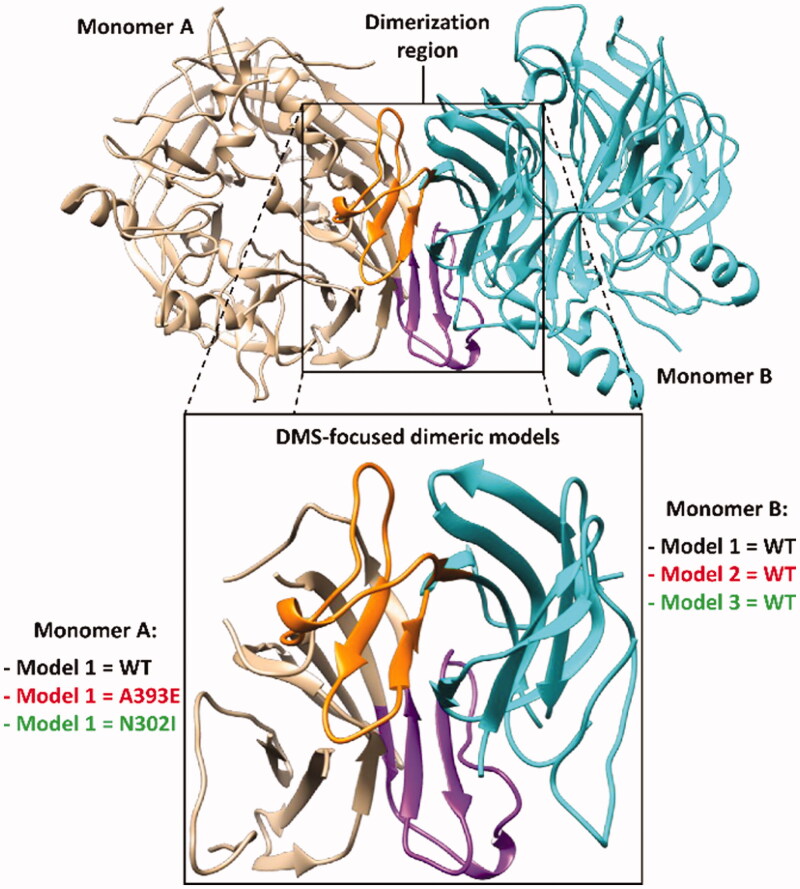
Structure of the DMS-focussed dimeric system compared to the full RPE65 dimer (PDB code 3FSN). The two monomers of both systems are shown in beige and cyan, with the corresponding DMS coloured purple and orange, respectively.

In the attempt of further improving the reliability of our MD-based strategy for evaluating the potential deleterious effect of missense mutations on the *h*RPE65 dimerisation region, we tried to increase the complexity of our model. Our aim was to obtain a more realistic representation of the *h*RPE65 dimeric system, which could also take into account the contribute of the mutual presence of the two monomers in the evaluation of the impact of the missense mutation on local folding and conformation stability of the DMS region of *h*RPE65. For this reason, by combining the WT and mutated *h*RPE65 models employed in the previous MD simulations, three different dimeric systems reproducing the bound dimerisation domains of both *h*RPE65 monomers were generated: a WT-WT dimeric system (Model 1) and two mutant-WT dimeric systems, including either A393E (Model 2) or N302I (Model 3) mutations in a single monomer (Figure 4). All systems were subjected to the same MD simulations using the HMR scheme employed for the monomeric systems. However, due to the higher computation time required for the simulation of the dimeric models compared to the original monomeric ones, the total simulation time was restricted to 3 µs. After the simulations, all models were subjected to the same analyses already performed on the monomeric systems. In particular, we focussed our attention on monomer A of the dimeric models, which was subjected to mutations in Models 2 and 3 (Figure 4).

**Figure 5.**
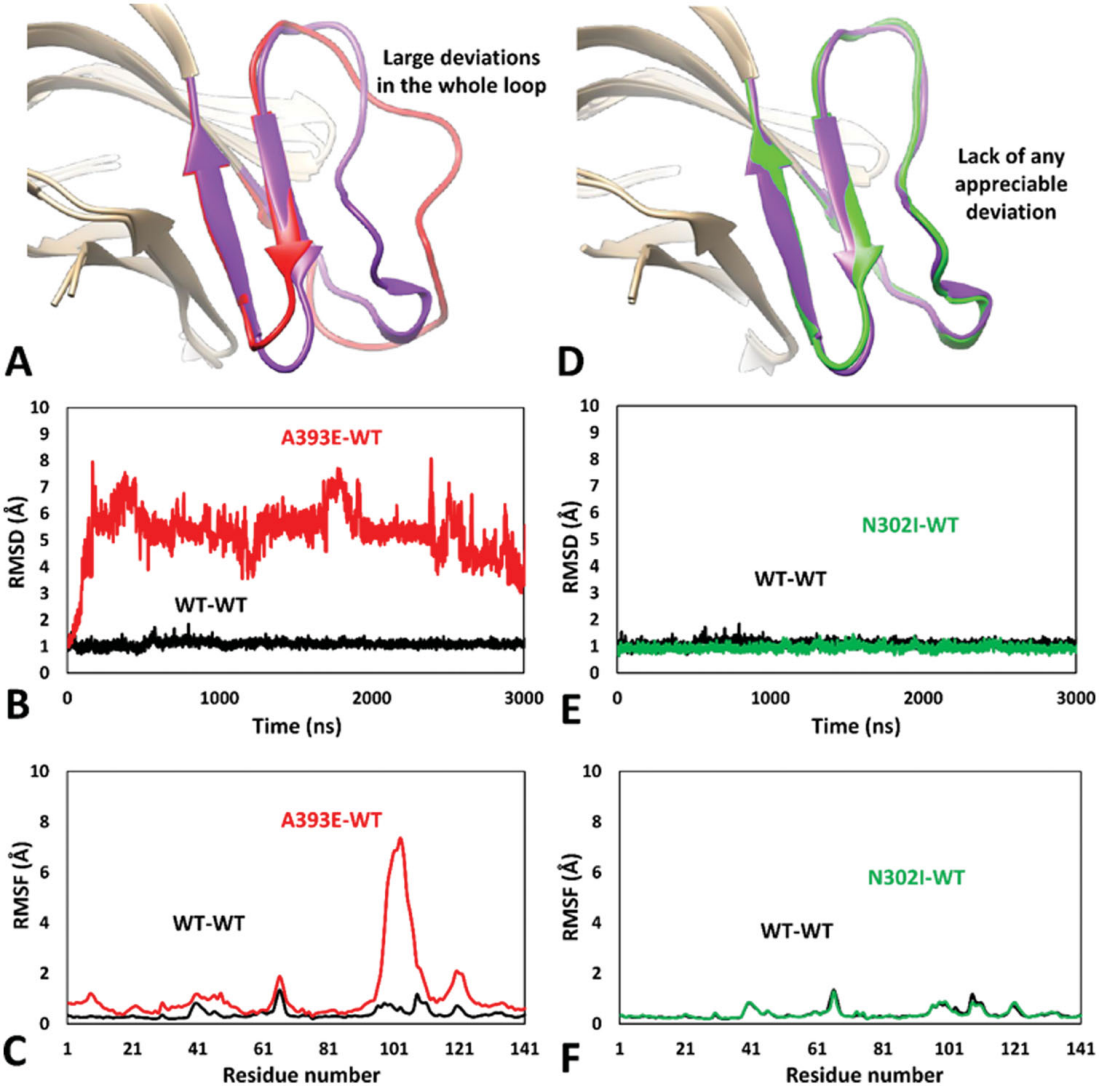
MD results obtained for the dimeric A393E-WT and N302I-WT models of *h*RPE65. (A) Average structures of A393E and WT monomers obtained from the last µs of MD simulation; the DMS of the two systems is respectively coloured red and purple. (B) RMSD of the DMS α carbons during 3 µs of MD simulation obtained for A393E monomer compared to WT. (C) RMSF of the whole A393E monomer α carbons compared to WT. (D) Average structures of N302I and WT monomers obtained from the last µs of MD simulation; the DMS of the two systems is respectively coloured green and purple. (E) RMSD of the DMS α carbons during 3 µs of MD simulation obtained for N302I monomer compared to WT. (F) RMSF of the whole N302I monomer α carbons compared to WT.

As expected, the WT-WT dimeric model showed results that were fully comparable with those obtained for the monomeric WT system, with an average RMSD of the DMS α carbons around 1 Å and maximum RMSF values of all α carbons below 1.5 Å (Figure 5 and Supplementary Figure S1). Consistently, the minimised average structure of the WT system in the WT-WT dimer obtained from the last µs of MD simulation was found to be fully comparable with that derived from the monomeric system (Supplementary Figure S2). Despite the reduced simulation time employed for the MD studies of the dimeric models, the results proved the reliability of the MD protocol in confirming and improving the results obtained using the monomeric models of the *h*RPE65 variants. In fact, the DMS of A393E variant within the A393E-WT model was found to undergo a fast unfolding process, leading to a remarkable deviation from the native conformation (Figure 5(A)) since the very beginning of the MD simulation; this was highlighted by the RMSD of its DMS α carbons that reached values around 5–6 Å after less than 0.2 µs of MD (Figure 5(B)), which were averagely maintained for the whole simulation (mean RMSD = 5.3 Å). By comparing the average structure obtained for the mutated A393E monomer with that obtained for the WT, it is possible to check that almost the whole DMS loop of the mutant presents large conformational alterations with respect to the WT and appreciable deviations from the native conformation are also observed within the DMS β-sheet (Figure 5(A)). In agreement with these observations, the RSMF of the α carbons of the whole mutated monomer showed a very high peak (above 7 Å) around the DMS loop residues, but also an appreciable increase in many other residues of the system (Figure 5(C)). The MD simulation results obtained for the N302I-WT system allowed to clearly confirm the harmless impact of N302I variant. In fact, the N302I monomer within the mutated dimer substantially showed the same results obtained for the native system. As shown in Figure 5(D), the minimised average structure of the N302I monomer obtained from the last µs of simulation was found to be perfectly superimposable to that of the WT monomer in all regions of the DMS and, in general, in all portions of the model. In fact, the DMS α carbons of the mutant showed RMSD values that were steadily kept around 1 Å for the whole MD simulation, as observed for the WT monomer (Figure 5(E)). Moreover, the RMSF plot obtained for the α carbons of the whole mutated monomer was almost indistinguishable from that related to the WT monomer (Figure 5(F)), which confirmed the lack of any appreciable deviation from the native conformation during the whole MD simulation.

Based on these results, the dimeric approach demonstrated to constitute a successful implementation of our DMS-focussed MD-based evaluation strategy of *h*RPE65 missense mutations, providing a confirmation of both the robustness of our MD protocol and the reliability of the potential pathogenicity predictions that can be derived from the obtained structure-based results. In fact, the dimeric DMS-focussed models allowed to speed up the occurrence of the deleterious effects determined by A393E mutation on local protein folding and conformation stability, as well as to eliminate the very small deviation observed in few loop residues of the monomeric model of N302I variant.

In conclusion, we developed an *in silico* protocol based on MD simulations aimed at evaluating the potential pathogenic effect of *h*RPE65 missense variants. Our strategy focussed on analysing the dimerisation domain of the protein, including the DMS and the surrounding region, by using DMS-focussed *h*RPE65 models. The two DMS-focussed MD-based approaches herein reported, employing both monomeric and dimeric models of WT and mutant *h*RPE65, were found to represent profitable tools for analysing the potential deleterious impact of missense variants on local folding and conformation stability around the dimerisation domain of the protein. Such approaches may thus be used either alone or better in combination with each other, with the aim of deriving solid structure-based clues about the potential pathogenicity of *h*RPE65 variants. The herein developed *in silico* protocols can be readily applied for the analysis of all other *h*RPE65 variants characterised by missense mutations localised within the *h*RPE65 dimerisation domain. In particular, our strategy can be applied for obtaining preliminary information about the potentially deleterious effect of at least 10 missense mutations lacking a clear pathogenicity classification or labelled as variants of uncertain significance (VUS) therein located. A more refined interpretation of VUS may have a significant clinical impact, especially in patients showing a retinal phenotype strongly suggesting to be RPE65-related but carrying RPE65 VUS. Such patients represent very challenging cases as their phenotype seems particularly suitable for receiving the treatment, but they cannot access it unless the pathogenicity of the RPE65 variants is demonstrated. The results may support possible reclassifications of *h*RPE65 missense variants in relation to pathological phenotypes such as RP, LCA and SECORD^30^, and may help clinicians to better evaluate the eligibility for gene therapy of patients diagnosed with such mutations. Moreover, our domain-focussed MD-based protocol for analysing the structural impact of *h*RPE65 missense mutations may be extended to other regions of the protein in order to expand the applicability domain of our strategy and allow the evaluation of the potential pathogenicity of *h*RPE65 VUS localised in different structural contexts, thus paving the way for the development of an exhaustive approach enabling the analysis of all possible of *h*RPE65 VUS. Finally, it’s worth highlighting that our approach may be profitably applied for evaluating the impact of missense mutations on other protein targets whose aberrant function and/or expression is implied in the development of inherited diseases. In particular, proteins characterised by the presence of distinct structural domains would be most suitable for our approach, since the effect of local folding and conformation stability of specific protein regions could be analysed through microseconds-long MD simulations of domain-focussed systems, as performed for the dimerisation region of *h*RPE65.

## Supplementary Material

Supplemental Material

## References

[1] Liu X, Chen J, Liu Z, et al. Potential therapeutic agents against retinal diseases caused by aberrant metabolism of retinoids. Invest Ophthalmol Vis Sci 2016;57:1017–30. 26962698 10.1167/iovs.15-18429

[2] Choi EH, Daruwalla A, Suh S, et al. Retinoids in the visual cycle: role of the retinal G protein-coupled receptor. J Lipid Res 2021;62:100040. 32493732 10.1194/jlr.TR120000850 PMC7910522

[3] Kiser PD, Zhang J, Badiee M, et al. Catalytic mechanism of a retinoid isomerase essential for vertebrate vision. Nat Chem Biol 2015;11:409–15. 25894083 10.1038/nchembio.1799 PMC4433804

[4] Moiseyev G, Crouch RK, Goletz P, et al. Retinyl esters are the substrate for isomerohydrolase. Biochemistry 2003;42:2229–38. 12590612 10.1021/bi026911y

[5] Kiser PD. Retinal pigment epithelium 65 kDa protein (RPE65): an update. Prog Retin Eye Res 2022;88:101013. 34607013 10.1016/j.preteyeres.2021.101013 PMC8975950

[6] Uppal S, Liu T, Poliakov E, et al. The dual roles of RPE65 S-palmitoylation in membrane association and visual cycle function. Sci Rep 2019;9:5218. 30914787 10.1038/s41598-019-41501-w PMC6435699

[7] Poliakov E, Uppal S, Rogozin IB, et al. Evolutionary aspects and enzymology of metazoan carotenoid cleavage oxygenases. Biochim Biophys Acta – Mol Cell Biol Lipids 2020;1865:158665. 32061750 10.1016/j.bbalip.2020.158665 PMC7423639

[8] Kiser PD, Farquhar ER, Shi W, et al. Structure of RPE65 isomerase in a lipidic matrix reveals roles for phospholipids and iron in catalysis. Proc Natl Acad Sci USA 2012;109:E2747–56. 23012475 10.1073/pnas.1212025109 PMC3478654

[9] Wright CB, Chrenek MA, Feng W, et al. The Rpe65 rd12 allele exerts a semidominant negative effect on vision in mice. Invest Ophthalmol Vis Sci 2014;55:2500–15. 24644049 10.1167/iovs.13-13574 PMC3993890

[10] Sallum JMF, Kaur VP, Shaikh J, et al. Epidemiology of mutations in the 65-kDa Retinal Pigment Epithelium (RPE65) gene-mediated inherited retinal dystrophies: a systematic literature review. Adv Ther 2022;39:1179–98. 35098484 10.1007/s12325-021-02036-7 PMC8918161

[11] Prado DA, Acosta-Acero M, Maldonado RS. Gene therapy beyond luxturna: a new horizon of the treatment for inherited retinal disease. Curr Opin Ophthalmol 2020;31:147–54. 32175942 10.1097/ICU.0000000000000660

[12] Russell S, Bennett J, Wellman JA, et al. Efficacy and safety of voretigene neparvovec (AAV2-hRPE65v2) in patients with RPE65-mediated inherited retinal dystrophy: a randomised, controlled, open-label, phase 3 trial. Lancet 2017;390:849–60. 28712537 10.1016/S0140-6736(17)31868-8 PMC5726391

[13] Sodi A, Banfi S, Testa F, et al. RPE65-associated inherited retinal diseases: consensus recommendations for eligibility to gene therapy. Orphanet J Rare Dis 2021;16:257. 34088339 10.1186/s13023-021-01868-4 PMC8176684

[14] Camp DA, Falabella P, Ciulla TA. RPE65 mutation-associated inherited retinal disease and gene therapies. Int Ophthalmol Clin 2021;61:125–32. 34584049 10.1097/IIO.0000000000000381

[15] Garafalo AV, Cideciyan AV, Héon E, et al. Progress in treating inherited retinal diseases: early subretinal gene therapy clinical trials and candidates for future initiatives. Prog Retin Eye Res 2020;77:100827. 31899291 10.1016/j.preteyeres.2019.100827 PMC8714059

[16] Maguire AM, Russell S, Chung DC, et al. Durability of voretigene neparvovec for biallelic RPE65-mediated inherited retinal disease. Ophthalmology 2021;128:1460–8. 33798654 10.1016/j.ophtha.2021.03.031

[17] Fokkema IFAC, Taschner PEM, Schaafsma GCP, et al. den Dunnen JT. LOVD v.2.0: the next generation in gene variant databases. Hum Mutat 2011;32:557–63. 21520333 10.1002/humu.21438

[18] Famiglietti ML, Estreicher A, Gos A, et al. Genetic variations and diseases in UniProtKB/Swiss‐Prot: the Ins and outs of expert manual curation. Hum Mutat 2014;35:927–35. 24848695 10.1002/humu.22594 PMC4107114

[19] Landrum MJ, Lee JM, Benson M, et al. ClinVar: public archive of interpretations of clinically relevant variants. Nucleic Acids Res 2016;44:D862–D868. 26582918 10.1093/nar/gkv1222 PMC4702865

[20] Stelzer G, Rosen N, Plaschkes I, et al. The GeneCards Suite: from gene data mining to disease genome sequence analyses. Curr Protoc Bioinforma 2016;54:1.30.1–1.30.33. 10.1002/cpbi.5 27322403

[21] Kiser PD, Golczak M, Lodowski DT, et al. Crystal structure of native RPE65, the retinoid isomerase of the visual cycle. Proc Natl Acad Sci 2009;106:17325–30. 19805034 10.1073/pnas.0906600106 PMC2765077

[22] Fiser A, Do RKG, Šali A. Modeling of loops in protein structures. Protein Sci 2000;9:1753–73. 11045621 10.1110/ps.9.9.1753 PMC2144714

[23] Case DA, Betz RM, Cerutti DS, et al. Amber 2016. San Francisco: University of California; 2016.

[24] Hopkins CW, Le Grand S, Walker RC, Roitberg AE. Long-time-step molecular dynamics through hydrogen mass repartitioning. J Chem Theory Comput 2015;11:1864–74. 26574392 10.1021/ct5010406

[25] Roe DR, Cheatham TE. PTRAJ and CPPTRAJ: software for processing and analysis of molecular dynamics trajectory data. J Chem Theory Comput 2013;9:3084–95. 26583988 10.1021/ct400341p

[26] Galvin JA, Fishman GA, Stone EM, Koenekoop RK. Evaluation of genotype-phenotype associations in leber congenital amaurosis. Retina 2005;25:919–29. 16205573 10.1097/00006982-200510000-00016

[27] Sundaresan P, Vijayalakshmi P, Thompson S, et al. Mutations that are a common cause of Leber congenital amaurosis in northern America are rare in southern India. Mol Vis 2009;15:1781–7. 19753312 PMC2742639

[28] Pasadhika S, Fishman GA, Stone EM, et al. Differential macular morphology in patients with RPE65 -, CEP290 -, GUCY2D -, and AIPL1 -related leber congenital amaurosis. Investig Opthalmology Vis Sci 2010;51:2608–14. 10.1167/iovs.09-3734 PMC2868490 19959640

[29] Takahashi Y, Moiseyev G, Ma J. Identification of key residues determining isomerohydrolase activity of human RPE65. J Biol Chem 2014;289:26743–51. 25112876 10.1074/jbc.M114.558619 PMC4175317

[30] Motta F, Martin R, Porto F, et al. Pathogenicity reclassification of RPE65 missense variants related to leber congenital amaurosis and early-onset retinal dystrophy. Genes 2019;11:24. 10.3390/genes11010024 PMC7016655 31878136

