# Supplementary material for: Predicting potentially pathogenic effects of hRPE65 missense mutations: a computational strategy based on molecular dynamics simulations
Source: J Enzyme Inhib Med Chem. 2022 Jun 21;37(1):1765–72. doi: 10.1080/14756366.2022.2090547 (PMC9225791; doi:10.1080/14756366.2022.2090547)
Supplement: Supplemental Material [file IENZ_A_2090547_SM4572.pdf]

Supporting Information

Predicting potentially pathogenic effects of *h*RPE65 missense mutations: a computational approach based on molecular dynamics simulations

Giulio Poli,[1] Ivana Barravecchia,[1,2] Gian Carlo Demontis,[1] Andrea Sodi,[3] Alessandro Saba,[4] Stanislao Rizzo,[5,6,7] Marco Macchia,[1] Tiziano Tuccinardi[1,*]

[1]Department of Pharmacy, University of Pisa, Pisa, Italy, [2]Institute of Life Sciences, Scuola Superiore Sant'Anna, Pisa, Italy [3]Department of Neurosciences, Psychology, Drug Research and Child Health Eye Clinic, University of Florence, AOU Careggi, Florence, Italy, [4]Department of Surgical Pathology, Molecular Medicine and of the Critical Area, University of Pisa, Pisa, Italy, [5]Ophthalmology Unit, Fondazione Policlinico Universitario A. Gemelli IRCCS, Rome, Italy, [6]Catholic University Sacro Cuore, Rome, Italy, [7]Consiglio Nazionale delle Ricerche, Istituto di Neuroscienze, Pisa, Italy.

Address for correspondence: Tiziano Tuccinardi, Department of Pharmacy, University of Pisa, Via Bonanno 6, 56126 Pisa, Italy. E-mail: tiziano.tuccinardi@unipi.it

Table of Content

Figure S1. RMSD and RMSF results for the WT-WT dimeric system. Pag. S2

Figure S2. Minimized average structures obtained from the WT-WT system. Pag. S2

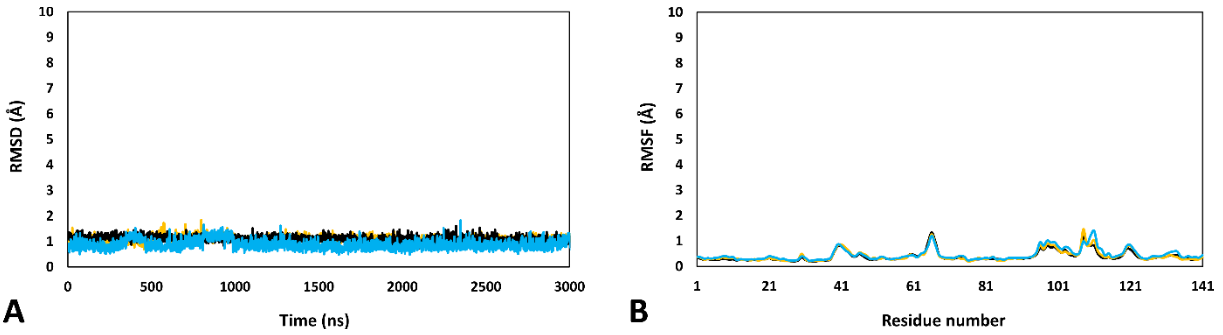

Figure S1. RMSD of DMS α carbons (A) and RMSF of all α carbons (B) obtained for both monomers of the WT-WT dimeric system (orange and black), compared to the monomeric WT system (cyan).

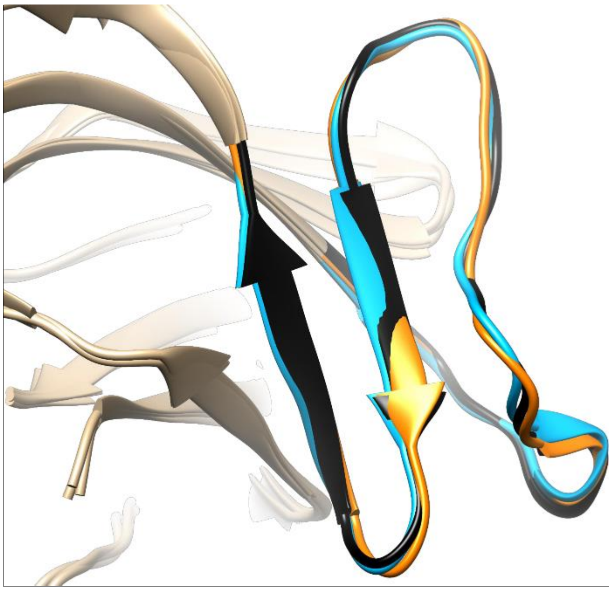

Figure S2. Minimized average structures of both monomers of the WT-WT dimeric system (orange and black) superimposed to that of the monomeric WT system (cyan).
